# Artificial Intelligence and Predictive Modelling for Precision Dosing of Immunosuppressants in Kidney Transplantation

**DOI:** 10.3390/ph19010165

**Published:** 2026-01-16

**Authors:** Sholpan Altynova, Timur Saliev, Aruzhan Asanova, Zhanna Kozybayeva, Saltanat Rakhimzhanova, Aidos Bolatov

**Affiliations:** 1 Department of Medical and Regulatory Affairs, Corporate Fund “University Medical Center”, Astana 010000, Kazakhstan; venera.altynova@umc.org.kz; 2 Institute for Fundamental and Applied Medical Research, S.D. Asfendiyarov Kazakh National Medical University, Almaty 050000, Kazakhstan; 3 Department of Science, Corporate Fund “University Medical Center”, Astana 010000, Kazakhstan; 4 Clinical Academic Department of Internal Medicine, Corporate Fund “University Medical Center”, Astana 010000, Kazakhstan; 5 Clinical Academic Department of Paediatrics, Corporate Fund “University Medical Center”, Astana 010000, Kazakhstan; 6 Shenzhen University Medical School, Shenzhen University, Shenzhen 518060, China

**Keywords:** kidney transplantation, calcineurin inhibitors, antimetabolites, mTOR inhibitors, drug monitoring, artificial intelligence (AI), machine learning, Bayesian pharmacokinetic

## Abstract

Optimizing immunosuppressant dosing presents significant challenges in kidney transplantation due to narrow therapeutic ranges and considerable inter-patient pharmacokinetic differences. Emerging strategies for precision dosing, encompassing Bayesian population pharmacokinetic models, pharmacogenomic integration, and artificial intelligence algorithms, aim to enhance drug monitoring by moving beyond traditional trough-based approaches. This review critically assesses available evidence for predictive dosing models targeting immunosuppressants, including calcineurin inhibitors, antimetabolites, and mTOR inhibitors in kidney transplant patients. Available observational and simulation studies demonstrate substantial methodological diversity, with Bayesian PopPK-guided strategies showing 15–35% better target exposure achievement compared to trough-based monitoring. The absence of pooled estimates precludes a precise summary effect size, and evidence from randomized controlled trials remains limited. Machine learning models, particularly for tacrolimus, frequently reduced prediction error relative to traditional regression approaches, but substantial heterogeneity in study design, outcome definitions, and external validation limits quantitative synthesis. Hybrid Bayesian–AI frameworks and explainable AI tools show conceptual promise but are largely supported by proof-of-concept studies rather than reproducible clinical implementations. Overall, Bayesian pharmacokinetic modelling represents the most mature and clinically interpretable approach for precision dosing in transplantation, whereas AI-driven and hybrid systems remain investigational. Key gaps include the need for standardized reporting, rigorous risk-of-bias assessment, prospective validation, and clearer regulatory and implementation pathways to support safe and equitable clinical adoption.

## 1. Introduction

Success in kidney transplantation hinges on accurate immunosuppressant dosing amid tight safety margins and variable drug levels in patients. Despite decades of AI research, routine care still relies mainly on after-the-fact changes using trough-level checks. For individuals with end-stage renal disease, kidney transplantation represents the optimal therapeutic option, offering superior survival rates, enhanced quality of life, and better economic efficiency relative to long-term dialysis treatment [1,2]. Despite these benefits, sustained graft function and patient survival depend heavily on the appropriate use of immunosuppressive medications following transplantation [3,4].

Kidney transplantation yields superior patient survival (97–98% at 1–5 years) and graft function compared to dialysis for end-stage renal disease in Kazakhstan, where resource constraints amplify dialysis morbidity [5]. However, stable immunosuppressant exposure proves elusive amid marked pharmacokinetic variability. In Central Asian cohorts, CYP3A51 expresser prevalence reaches ~30–50% (Uygur/Kazakh populations), necessitating 1.5–2-fold higher tacrolimus doses versus CYP3A53/*3-dominant Europeans, as evidenced by slower clearance in poor metabolizers.

Through monitoring, the standard in Kazakh centres misses dynamic AUC fluctuations during early post-transplant inflammation and polypharmacy, common in comorbid recipients, yielding 30–50% prediction errors. Predictive dosing tools enhance target attainment by 11–39% and could reduce rejection risk by ~20–25% through faster stabilization, although Western-centric validation limits generalizability to diverse ethnicities, such as Kazakh. Ethnically tuned Bayesian or AI models are essential for equitable application [6,7].

Treatment goals focus on curbing immune attacks on the graft without over-suppression that causes toxicity or infections. This equilibrium proves difficult as these drugs show tight efficacy ranges and wide PK/PD (pharmacokinetics and pharmacodynamics) differences across patients.

Key drug classes used in post-transplant immunosuppressive regimens—including calcineurin inhibitors (tacrolimus and cyclosporine), antimetabolites (mycophenolate mofetil and azathioprine), and mammalian target of rapamycin (mTOR) inhibitors (sirolimus and everolimus)—demonstrate substantial variability within and between individuals [8]. This variability reflects the combined influence of genetic determinants (such as polymorphisms in CYP3A5, ABCB1, and UGT1A9), patient-specific characteristics (including age, sex, and body composition), comorbid conditions, concomitant medications, and evolving physiological states during the post-transplant period [9,10]. Consequently, identical dosing strategies can produce highly divergent systemic drug exposures, increasing the risk of either insufficient immunosuppression and graft rejection or excessive exposure leading to nephrotoxicity, neurotoxicity, and metabolic adverse effects.

Therapeutic drug monitoring (TDM), particularly the assessment of trough drug concentrations (C_0_), has traditionally served as the principal method for guiding immunosuppressant dose adjustments. Although TDM remains integral to transplant pharmacotherapy, it provides only a limited, static representation of a complex and dynamic biological process [11]. Standard population-based dosing approaches often lack the ability to reflect individual PK trajectories or account for nonlinear behaviour, especially in the early post-transplant phase when absorption, metabolism, and distribution are rapidly changing [12]. Furthermore, reliance on empirical dose modification and clinician experience can introduce variability in practice and reduce consistency across transplant centres.

Recent progress in pharmacometric modelling, computational sciences, and biomedical informatics has facilitated the development of more individualized dosing strategies. In this context, artificial intelligence (AI) and machine learning (ML) methodologies have gained increasing attention due to their capacity to process large, multidimensional datasets derived from pharmacogenomic testing, laboratory measurements, electronic health records (EHRs), and longitudinal drug concentration profiles [13]. By identifying latent patterns and nonlinear interactions among covariates, AI algorithms can predict individualized drug responses and optimize dose selection in real time.

In parallel, Bayesian pharmacokinetic modelling has emerged as a complementary and mechanistically grounded approach to personalized dosing. Bayesian frameworks dynamically integrate prior population-level information with individual patient data to generate posterior estimates of drug exposure, allowing iterative refinement of dosing decisions as new observations become available [14]. The integration of Bayesian methods with AI-driven models offers the potential to combine physiological interpretability with advanced pattern recognition, resulting in hybrid systems that balance predictive performance with clinical transparency.

Building on these methodological advances, several digital clinical decision-support platforms have been developed to translate predictive dosing concepts into routine practice. Software solutions such as InsightRx Nova™, MW/Pharm++, and AID-Tac incorporate patient-specific variables, TDM results, and pharmacogenomic data to provide automated dosing recommendations at the point of care [15]. While improved exposure control is biologically plausible as a contributor to better clinical outcomes, direct evidence linking AI- or model-informed dosing strategies to reduced rejection or mortality remains heterogeneous, and results from prospective randomized trials have been variable.

Despite growing interest in AI-driven dosing platforms such as InsightRx Nova™ and AID-Tac, evidence supporting their impact on hard clinical endpoints remains mixed. While several studies report improved attainment of therapeutic drug concentrations and reduced time to target range, randomized controlled trials demonstrating consistent benefits in graft survival, rejection rates, or long-term patient outcomes are limited. In some settings, neutral effects have been observed, highlighting the influence of local workflows, data quality, and clinician engagement on system performance. Moreover, most currently available platforms function as clinical decision-support tools rather than autonomous systems, and their regulatory classification varies across jurisdictions. Challenges related to algorithm transparency, external validation, population-specific generalizability, and seamless integration into electronic health record infrastructures continue to limit widespread adoption. Consequently, these tools should be viewed as adjuncts to, rather than replacements for, clinical expertise, pending further prospective validation.

Together, advances in AI, pharmacometrics, and digital health signal a transition from reactive dose adjustment toward proactive precision dosing in kidney transplantation. Predictive modelling enables clinicians to anticipate deviations in drug exposure before adverse outcomes occur, thereby supporting improved graft longevity and patient safety. Nevertheless, widespread adoption of these approaches will require continued efforts to enhance model interpretability, ensure data harmonization, validate clinical effectiveness, and integrate decision-support systems within existing healthcare infrastructures.

This manuscript is intended as a critical narrative review with systematic elements, rather than a tutorial or opinion piece. The scope is limited to predictive and model-informed precision dosing approaches for immunosuppressive therapy in kidney transplantation, with a specific focus on Bayesian population pharmacokinetic modelling, machine learning-based prediction, pharmacogenomic integration, and hybrid Bayesian–AI frameworks. Emphasis is placed on evaluating the strength, transparency, and clinical relevance of the available evidence rather than providing an exhaustive catalogue of published models or software platforms. The target audience includes transplant clinicians, clinical pharmacologists, pharmacists, and pharmacometricians involved in therapeutic drug monitoring and dose individualization, as well as researchers developing or validating predictive dosing systems. Accordingly, the review prioritizes clinically actionable outcomes (e.g., target exposure attainment, prediction error, and feasibility of implementation), methodological rigour (study design, validation, and risk of bias), and translational considerations such as regulatory oversight, workforce training, and equity of access. By delineating evidentiary maturity and unresolved gaps, this review aims to support informed clinical adoption and to guide future research priorities in precision immunosuppression.

## 2. Review Methodology

This article is a narrative review informed by a structured literature search. To balance historical context with recent innovation, the primary search was conducted in PubMed, Scopus, and Web of Science for the period January 2010 through December 2024. This timeframe was selected to capture the modern era of model-informed precision dosing and the rapid emergence of AI/ML applications in transplantation pharmacotherapy. A supplemental forward-citation search and alert monitoring were employed to identify key early-2025 publications relevant to the core themes of the review. The search strategy combined controlled vocabulary (e.g., MeSH terms) and keywords related to: (“kidney transplantation”) AND (“immunosuppressive agents” OR “tacrolimus”) AND (“artificial intelligence” OR “machine learning” OR “Bayesian” OR “precision dosing” OR “pharmacokinetic model”). The reference lists of included systematic reviews and key articles were hand-searched to identify additional foundational studies, including those published prior to 2010, to ensure coverage of seminal clinical trials and methodological papers. Priority was given to peer-reviewed original research, systematic reviews, and methodological studies reporting on model development, validation, or clinical implementation. Given the substantial heterogeneity in study designs, populations, outcome definitions, and analytical methods across the identified literature, a formal meta-analysis to produce pooled effect estimates with confidence intervals was not feasible. Therefore, quantitative performance improvements are reported as ranges observed across studies to illustrate the landscape of findings, not as statistically pooled results. Only English-language publications were included.

## 3. Variability and the Need for Precision Dosing

Handling PK/PD differences, across patients and within the same patient over time, poses a core challenge in transplant management. Standard protocols still result in uneven drug exposure from the same doses among recipients [16]. This variability arises from a complex interplay of genetic, physiological, environmental, and behavioural factors that influence absorption, distribution, metabolism, and elimination of these agents. Understanding and managing this heterogeneity is essential to maintain the delicate balance between preventing graft rejection and avoiding drug-induced toxicity [17].

Gene differences in drug metabolism and transport drive much of the dosing variation. Tacrolimus and cyclosporine mainly rely on CYP3A5/CYP3A4 for breakdown and ABCB1 for cellular movement [18,19] (Table 1). Polymorphisms in these genes have well-documented clinical consequences. Carriers of the CYP3A5 3/3 genotype, who lack functional enzyme expression, exhibit significantly reduced clearance and higher systemic exposure to tacrolimus, requiring lower doses to prevent toxicity [20]. Conversely, individuals expressing functional CYP3A5 alleles (*1 carriers) metabolize tacrolimus more rapidly and need higher maintenance doses to achieve therapeutic concentrations [21]. Similarly, ABCB1 variants such as C3435T and G2677T/A influence intestinal absorption and intracellular drug distribution, potentially affecting both efficacy and adverse reactions [22]. Other immunosuppressants are also subject to pharmacogenomic influence; for example, polymorphisms in UGT1A9 and SLCO1B1 alter the metabolism of mycophenolate mofetil, while CYP3A4 and ABCB1 variants affect the pharmacokinetics of mTOR inhibitors like sirolimus and everolimus [23]. Although pharmacogenetic testing offers a mechanistic basis for individualized dosing, its routine clinical implementation remains limited, underscoring the need for predictive approaches capable of integrating genetic information with dynamic clinical data.

This genetic heterogeneity has profound implications for model development and clinical implementation. The population frequency of functional alleles, such as CYP3A5*1, exhibits dramatic geographical and ethnic stratification. For example, the CYP3A5*1 allele frequency exceeds 50% in many populations of African ancestry but is below 15% in many East Asian and European populations. Consequently, the average tacrolimus dose requirement for a population with high CYP3A5*1 frequency can be substantially higher than for a population where the non-functional *3 allele is predominant. Predictive dosing models trained on data from one ethnic group without accounting for these underlying genetic differences risk systematic bias when applied to another, leading to consistent under- or over-dosing. Therefore, the external validity of any pharmacogenomics-informed model is intrinsically linked to the genetic representativeness of its training cohort, highlighting a critical need for diverse, multi-ethnic data in model development.

Physiological and clinical factors further contribute to the variability in drug disposition. Age, sex, body composition, liver and kidney function, haematocrit, and albumin levels all affect the pharmacokinetic profile of immune suppressants. Pediatric and elderly recipients, for instance, often require dose adjustments due to differences in hepatic enzyme activity, protein binding, and distribution volumes. Renal and hepatic dysfunction, which are common during the peri-transplant period, can profoundly alter clearance rates. Drug–drug interactions also play a critical role; co-administration of CYP3A inhibitors such as azole antifungals, macrolides, or calcium channel blockers can dramatically increase tacrolimus concentrations, whereas enzyme inducers such as rifampicin or phenytoin can lead to subtherapeutic exposure [24,25]. Moreover, systemic inflammation and infection can modulate the expression of metabolic enzymes and transporters, resulting in unpredictable shifts in drug clearance and tissue distribution.

Behavioural and environmental factors add yet another dimension of complexity. Variability in medication adherence, dosing schedules, and dietary habits can significantly alter systemic exposure. Non-adherence, whether intentional or due to complex medication regimens, is one of the most common causes of late allograft dysfunction and rejection [26]. Circadian variation and food intake influence the absorption of tacrolimus, with reduced bioavailability observed when taken with high-fat meals [27]. Lifestyle and environmental factors, such as the use of herbal supplements or nicotine, can further modulate enzyme activity, creating additional variability that complicates dose optimization [16] (Table 1).

Analytical and methodological limitations in therapeutic drug monitoring compound these challenges. It is important to distinguish between pharmacokinetic exposure metrics and model performance metrics used in predictive modelling. In pharmacokinetics, the area under the concentration–time curve (AUC_0_–_24_) represents a quantitative measure of total systemic drug exposure and is directly linked to clinical outcomes such as rejection and toxicity. In contrast, in machine learning model evaluation, the area under the receiver operating characteristic curve (AUC-ROC) is a statistical performance metric that reflects a model’s ability to discriminate between predefined outcome classes and does not represent drug exposure. Machine learning models for immunosuppressant dosing are typically evaluated using error-based metrics such as root mean squared error (RMSE) and mean absolute error (MAE) for continuous outcomes (e.g., predicted concentrations or doses), as well as classification metrics including accuracy, precision, recall, and F1-score when categorical targets are used. Clear differentiation between pharmacokinetic endpoints and predictive performance metrics is essential for the appropriate interpretation and comparison of AI-based dosing models. Moreover, differences in analytical techniques, such as immunoassays versus LC–MS/MS, can yield inconsistent results and contribute to uncertainty in dose adjustments (Table 1).

To date, most studies evaluating predictive dosing platforms in transplantation have focused on pharmacokinetic endpoints or short-term process measures. Comprehensive randomized trials and formal health-economic analyses are still needed to determine whether these technologies translate into durable clinical benefits or cost-effectiveness advantages at the system level.

The clinical implications of these multifactorial variations are profound. Underexposure to immune-suppressants can precipitate acute or chronic rejection, jeopardizing graft survival, while overexposure leads to nephrotoxicity, hypertension, metabolic disorders, and increased susceptibility to infections and malignancies [28]. Even minor deviations from the therapeutic window can accumulate over time, affecting long-term graft function and patient morbidity. The conventional “one-dose-fits-all” paradigm is thus inadequate in the context of such individualized and dynamic pharmacology [29].

These challenges underscore the imperative for precision dosing, an approach that moves beyond empirical, and population-based dosing algorithms toward data-driven, individualized therapy. Precision dosing seeks to tailor drug selection and dosing in real time according to each patient’s genetic background, physiological state, and evolving clinical parameters. However, the complexity and nonlinearity of these relationships exceed the capacity of traditional statistical methods. Consequently, artificial intelligence and predictive modelling have emerged as powerful tools to address this challenge. By leveraging large-scale datasets, integrating pharmacogenomic and clinical information, and continuously learning from real-world data, AI-based systems and Bayesian pharmacokinetic models enable clinicians to anticipate deviations in drug exposure before they manifest clinically. This transition, from reactive dose adjustment to proactive prediction, marks a fundamental shift in transplant pharmacotherapy, paving the way for safer, more effective, and truly personalized immunosuppressive management (Table 1).

AI and ML approaches address pharmacokinetic variability by simultaneously integrating heterogeneous data sources that influence drug exposure. These models incorporate pharmacogenomic variables (e.g., CYP3A5 genotype), static clinical covariates (age, body weight, comorbidities), dynamic laboratory parameters (serum creatinine, haematocrit), concomitant medications, and longitudinal therapeutic drug monitoring data. By modelling nonlinear relationships and higher-order interactions among these variables, AI systems can capture sources of variability that are poorly represented in traditional rule-based or linear dosing algorithms. In practical terms, this results in improved predictive accuracy of drug concentrations or dose requirements, reduced inter- and intra-patient variability, faster attainment of target exposure ranges, and fewer corrective dose adjustments during the unstable early post-transplant period.

## 4. Machine Learning Approaches for Immunosuppressant Dose Prediction

Recent years have witnessed accelerated development of machine learning applications for immunosuppressant dose prediction, facilitated by growing access to comprehensive clinical data repositories and enhanced computational capabilities. Some studies have demonstrated that ML-based models outperform conventional regression or rule-based dosing algorithms in predicting tacrolimus concentrations and dose requirements, particularly when pharmacogenomic and longitudinal data are incorporated [30,31,32,33]. Moreover, early clinical implementations suggest that these approaches may reduce time to therapeutic range and dosing variability in real-world transplant populations [34,35].

ML tools combine genetic, clinical, and TDM inputs to forecast tacrolimus levels, though results suffer from biases in training datasets. Models developed primarily on Western, predominantly Caucasian datasets show markedly reduced accuracy in underrepresented populations, with mean absolute error (MAE) frequently exceeding 30% in validation studies. Ensemble methods such as random forests and XGBoost are effective in capturing nonlinear interactions, such as between genotype and haematocrit, improving trough concentration prediction by 20–30% over linear regression (MAE 1.8–2.2 vs. 2.7–3.1 ng/mL) in genotype-stratified analyses. However, advanced approaches like recurrent neural networks (RNNs) for longitudinal TDM remain investigational, limited by inconsistent endpoints, such as trough versus AUC measurements, and poor reproducibility, with inter-study variance often exceeding 25%.

In this context, the review by Bayanova, Zhenissova, and colleagues provides a clinically oriented synthesis of established pharmacogenetic principles guiding tacrolimus therapy [10]. It emphasizes the pivotal roles of CYP3A5, CYP3A4, and ABCB1 (MDR1) polymorphisms in tacrolimus metabolism and transport, with the CYP3A5 genotype (*1 vs. *3) recognized as the strongest predictor of dosing requirements. The authors advocate for genotype-informed dosing, particularly CYP3A5 testing, to accelerate therapeutic target attainment, reduce toxicity, and personalize immunosuppression. As a narrative review, it consolidates rather than extends evidence, but its clinically focused perspective and multidisciplinary authorship support the translation of pharmacogenetics into transplant practice, reinforcing the need for genetic integration to overcome the limitations of conventional and AI-driven dosing approaches alike.

In general, ML methodologies differ fundamentally from traditional regression-based approaches by capturing nonlinear, complex relationships among multiple clinical, genetic, and biochemical factors, rather than assuming simple linear associations [36]. ML models for immunosuppressant dosing are typically trained on large datasets comprising thousands of patient records, including demographic information, laboratory values, concomitant medications, genotypes, and time-stamped therapeutic drug monitoring (TDM) results [37] (Table 2). By analyzing these multidimensional data, ML algorithms can detect subtle patterns and interactions that would be impossible to identify through human reasoning or traditional statistics. For tacrolimus, the most widely studied agent, algorithms such as random forests, gradient boosting machines, support vector machines, and artificial neural networks have been successfully used to predict optimal doses or trough concentrations based on routine clinical variables and pharmacogenetic markers such as the CYP3A5 genotype [38,39]. These models learn to infer how each variable contributes to drug exposure, enabling dynamic and individualized dose recommendations.

It is crucial to contextualize these performance improvements. The reported ranges (e.g., RMSE reductions of 15–30%) stem from heterogeneous, single-centre, retrospective studies. A formal meta-analysis to produce a pooled effect size is not currently feasible due to profound methodological diversity in model architectures, outcome definitions (predicted dose vs. concentration), validation strategies, and patient populations. This heterogeneity itself is a major evidence gap, underscoring the need for standardized evaluation frameworks and prospective, multi-centre trials to generate reliable, generalizable effect estimates.

Among the most prominent examples is the development of ensemble learning models that integrate multiple weak learners to achieve robust predictions. Random forest algorithms, for instance, have been widely applied to tacrolimus dosing due to their ability to handle nonlinear relationships and complex feature interactions (Table 2). Studies comparing random forests with linear regression models have demonstrated that ML-based approaches yield lower prediction errors for tacrolimus trough concentrations and dose requirements, particularly when genotype data are included [40,41]. Gradient boosting algorithms such as XGBoost and LightGBM have shown similar or superior performance, offering advantages in handling imbalanced datasets and outliers commonly found in clinical pharmacokinetic data [42] (Table 2).

Artificial neural networks (ANNs) and deep learning architectures represent a more recent and powerful class of predictive models. These systems can automatically learn hierarchical representations of complex relationships between clinical inputs and pharmacokinetic outcomes without the need for explicit feature engineering [43]. Deep neural networks trained on comprehensive datasets of transplant recipients have demonstrated the capacity to capture nonlinear effects of multiple interacting variables, including liver function, haematocrit, serum creatinine, and concomitant medications. Some studies have applied recurrent neural networks (RNNs) and long short-term memory (LSTM) networks to sequential therapeutic monitoring data, allowing models to learn temporal dependencies and predict how drug concentrations evolve over time [44,45]. This temporal awareness is particularly valuable in the early post-transplant phase, where pharmacokinetics change rapidly due to physiological instability and interacting medications.

Recent advances have also explored the use of reinforcement learning (RL) and hybrid modelling approaches. Reinforcement learning algorithms simulate a decision-making process that continuously adapts drug-dosing strategies based on feedback from real-world outcomes, such as measured trough concentrations or clinical markers of rejection and toxicity [46]. By learning from sequential interactions, RL models can mimic the dynamic titration decisions made by clinicians but with enhanced consistency and precision. In pilot studies, these systems have shown promise in automatically recommending dose adjustments that keep patients within target therapeutic ranges, thereby reducing variability and minimizing the time required to achieve stable immunosuppression [47].

Another emerging approach involves hybrid models that combine mechanistic pharmacokinetic understanding with data-driven ML flexibility. In these models, traditional population pharmacokinetic (PopPK) or Bayesian frameworks provide physiological interpretability and parameter constraints, while ML components model residual variability unexplained by standard equations [48,49]. A growing body of work in other therapeutic areas provides concrete examples of hybrid and adaptive machine learning (ML) approaches that inform the potential path for transplant immunosuppressant dosing. These studies, while not transplant-specific, illustrate the methods, performance metrics, and validation strategies needed.

The study by Jasmine H. Hughes and Keizer (2021) [50] provides a seminal example of a true hybrid ML/pharmacokinetic (PK) model. In a large cohort of adult vancomycin patients, they used ML classifiers to identify instances where a patient was poorly described by the standard population PK model prior. In these cases, they applied “flattened priors” to reduce the model’s influence, allowing the individual’s data more weight. This hybrid ML/PK system, using factors like past prediction residuals as ML inputs, reduced overall prediction root mean squared error (RMSE) by 12–22% compared to standard maximum a posteriori (MAP) Bayesian estimation alone. This demonstrates a practical framework where ML optimizes the application of a mechanistic PK model.

Further work by Jasmine H. Hughes et al. (2020) [51] on pediatric vancomycin explored ‘continuous learning’, another key hybrid concept. They showed that starting with a published model and then iteratively refitting it with local patient data (a form of model “recalibration”) reduced prediction error by 2–13%. This highlights how hybrid systems can evolve, using local data streams to adapt a foundational model to a specific patient population, a highly relevant strategy for individual transplant centres.

Finally, Tang et al. (2023) demonstrated a powerful sequential hybrid approach for neonates [52]. They developed a CatBoost ML model to predict the initial vancomycin trough (\(C_0\)) ‘a priori’, which showed a 42.5% improvement in accuracy over a population PK model. After a trough measurement was obtained, a second CatBoost model predicted the area under the curve (AUC). This two-stage, ML-informed process significantly increased target attainment in virtual trials. This architecture is directly transferable to tacrolimus dosing, where an ML model could predict the initial dose or exposure, and a Bayesian model could then refine predictions using TDM data.

These studies provide the **methodological blueprint and performance benchmarks** (e.g., RMSE reductions of 12–22%, target attainment improvements) for hybrid systems. They demonstrate that hybrid models are not merely conceptual but can be implemented with measurable benefits in real-world dosing. However, they also reveal the current gap: **a directly analogous, fully validated hybrid ML/PK model for tacrolimus, cyclosporine, or mycophenolate is absent from the literature.** The cited work establishes the “how” and the “potential benefit,” but the transplant pharmacology field must now produce the specific “case study” where these methods are applied, externally validated, and shown to improve outcomes for kidney transplant recipients.

The studies by Hughes et al. and Tang et al. provide a methodological blueprint and performance benchmarks (e.g., RMSE reductions of 12–22%, target attainment improvements) for hybrid systems [50,52]. They demonstrate that hybrid models are not merely conceptual but can be implemented with measurable benefits in real-world dosing for agents like vancomycin. However, they also reveal the current critical gap: A directly analogous, fully validated hybrid ML/PK model for tacrolimus, cyclosporine, or mycophenolate in kidney transplantation is absent from the literature. The cited work establishes the ‘how’ and the ‘potential benefit,’ but the transplant pharmacology field must now produce the specific ‘case study’ where these methods are applied, externally validated, and shown to improve outcomes for kidney transplant recipients. Therefore, hybrid Bayesian-AI models remain an important investigational path rather than a current clinical reality in transplantation.

This integration of “white-box” pharmacological structure with “black-box” predictive learning has been shown to improve the accuracy and generalizability of tacrolimus and cyclosporine dosing models across diverse patient cohorts. For instance, hybrid PK–ML frameworks can use limited patient sampling to estimate PK parameters such as clearance and volume of distribution, while simultaneously refining predictions through adaptive learning from new patient data [48,50].

For clarity, population pharmacokinetic (PopPK) models describe typical drug disposition and variability within a patient population using mechanistic or semi-mechanistic parameters. Bayesian forecasting builds upon PopPK models by combining prior population estimates with individual patient observations to generate personalized predictions of drug exposure. In contrast, machine learning (ML) approaches rely on data-driven algorithms to model relationships between inputs and outcomes without explicitly specifying pharmacokinetic structure.

A conceptual comparison of population pharmacokinetic modelling, Bayesian forecasting, machine learning, and explainable artificial intelligence is provided in Table 2 to clarify their distinct roles in precision dosing.

Despite these advances, several challenges persist. One of the key issues is model generalizability across diverse populations. Many machine learning models are developed using data from single transplant centres or specific ethnic groups, which limits their applicability to other regions where pharmacogenomic profiles and clinical practices differ. For example, CYP3A5 allele frequencies vary substantially across populations, influencing dose requirements and predictive performance [53,54,55]. Addressing this limitation requires large, multicentre, and ethnically diverse datasets to ensure that AI-driven dosing tools can perform reliably in heterogeneous patient populations.

Equally important is the interpretability of machine learning models. Clinicians must understand how predictions are generated to trust and act upon them in critical therapeutic decisions. To address this, explainable AI (XAI) techniques such as SHAP (Shapley Additive Explanations) and LIME (Local Interpretable Model-Agnostic Explanations) are increasingly being incorporated into ML-based pharmacotherapy systems [56,57]. These methods can quantify the contribution of each variable, such as genotype, haematocrit, or co-administered drug, to the final dose prediction, providing transparency and clinical insight.

Explainable artificial intelligence (XAI) refers to a set of post hoc or intrinsic techniques designed to improve the interpretability and transparency of machine learning models. XAI does not constitute a standalone modelling framework but rather provides tools to elucidate how ML-based predictions are generated, particularly in clinical decision-support contexts. However, the practical application and evaluation of XAI in transplantation pharmacotherapy remain nascent. A critical distinction must be made between local interpretability, which explains an individual prediction (e.g., “this patient’s predicted high tacrolimus dose is primarily due to their CYP3A5 *1/*1 genotype and low hematocrit”), and global interpretability, which characterizes the overall behaviour of the model (e.g., “genotype is the most important feature across the entire patient cohort”). While tools like LIME excel at providing local, case-by-case explanations, they may not reveal consistent global model logic. SHAP can offer both, but requires careful clinical interpretation. In the transplant context, specific limitations emerge. First, the “explanations” provided are statistical attributions, not causal proofs, and may conflict with established pharmacokinetic principles, potentially eroding rather than building trust. Second, most evaluations of XAI in this field are technical demonstrations of feature importance; there is a stark lack of studies assessing whether these explanations actually improve clinician decision-making, reduce cognitive bias, or lead to better patient outcomes. Furthermore, for complex models like deep neural networks, even with XAI, the explanations themselves can be complex and difficult to integrate into a fast-paced clinical workflow. Therefore, while XAI is a necessary component for transparent AI, its current utility in routine transplant care is unproven, and its implementation must be accompanied by clinical validation and user-centred design to ensure explanations are both accurate and actionable.

Moreover, the integration of ML-driven dosing systems into real-world clinical workflows demands robust data pipelines, standardized reporting formats, and seamless interoperability with electronic health records (EHRs). Cloud-based platforms have begun to operationalize these concepts, embedding ML and Bayesian prediction algorithms into user-friendly digital interfaces that allow clinicians to input patient parameters and receive real-time dosing recommendations [58,59,60].

The convergence of advanced machine learning techniques, pharmacogenomic data, and real-time clinical information marks a transformative step toward precision dosing in transplantation. By integrating predictive analytics with clinical decision-support systems, AI-driven models offer the potential to reduce therapeutic variability, accelerate dose stabilization, and improve long-term graft outcomes. However, to achieve widespread clinical adoption, these tools must undergo rigorous prospective validation, regulatory evaluation, and ethical oversight to ensure their reliability, safety, and transparency. As the field progresses, collaborative efforts between transplant clinicians, pharmacologists, and data scientists will be crucial in transforming these computational innovations into tangible improvements in patient care.

## 5. Bayesian and Hybrid AI-Pharmacokinetic Modelling for Precision Dosing

Bayesian pharmacokinetic frameworks have become increasingly prominent for achieving precise dosing in transplantation settings. Unlike purely data-driven machine learning systems that may function as opaque “black boxes,” Bayesian methods maintain stronger connections to established pharmacological mechanisms while incorporating probabilistic reasoning [61]. They enable clinicians to integrate prior knowledge about population-level pharmacokinetic parameters with individual patient data to generate personalized dosing recommendations in real-time. This capacity for adaptive learning and continuous refinement makes Bayesian modelling uniquely suited for immunosuppressant therapy, where maintaining drug exposure within a narrow therapeutic window is essential and physiological conditions change dynamically over time.

Bayesian methodology follows a coherent principle of integrating established population-level pharmacokinetic knowledge (prior information) with individual patient data, including drug concentration measurements and clinical characteristics, to generate personalized parameter estimates (posterior distributions) for each patient [62]. These parameters, such as clearance, volume of distribution, and absorption rate constant, can then be used to predict future drug concentrations under different dosing regimens. Each new therapeutic drug monitoring (TDM) result further refines the posterior estimate, continuously updating the model as more data become available [63]. In this way, Bayesian forecasting acts as a dynamic feedback system that “learns” from each patient’s evolving pharmacokinetic profile, achieving individualized dosing precision that static equations or empirical adjustment cannot provide.

The appeal of Bayesian models in transplantation stems from their ability to provide reliable predictions even with limited sampling data, a major advantage in clinical settings where frequent blood draws are impractical or undesirable. Traditional non-compartmental or population-based pharmacokinetic methods require extensive sampling to accurately estimate the area under the concentration–time curve (AUC), whereas Bayesian approaches can infer this from just one or two concentration points when combined with robust prior models [64]. This efficiency is particularly valuable in pediatric, elderly, or critically ill transplant recipients, where patient burden and clinical instability often preclude dense sampling schedules.

In clinical practice, Bayesian pharmacokinetic models have been extensively applied to key immune-suppressants such as tacrolimus, cyclosporine, mycophenolate mofetil, and sirolimus. For tacrolimus, in particular, Bayesian forecasting has demonstrated superiority over traditional trough-based monitoring in achieving and maintaining target exposure [65]. Numerous studies have shown that Bayesian-guided dosing enables faster attainment of therapeutic AUC targets, reduces inter- and intra-patient variability, and minimizes both underexposure and overexposure-related complications [66,67]. For instance, multicentre evaluations have reported that patients managed with Bayesian dose prediction reach steady-state concentrations within target ranges more consistently than those dosed empirically, thereby reducing the risk of acute rejection in the early post-transplant period [68,69]. However, the majority of supporting evidence derives from observational and simulation studies. Pooled effect estimates from meta-analyses are absent due to significant methodological heterogeneity across trials. While Bayesian-guided dosing consistently improves target exposure attainment in these studies, high-quality randomized controlled trials with clinical endpoints (e.g., graft survival, rejection rates) remain sparse.

The predominance of observational and simulation data must be emphasized. While Bayesian-guided dosing consistently shows improved target exposure attainment in these studies, high-quality randomized controlled trials (RCTs) with clinical endpoints (e.g., biopsy-proven acute rejection, graft survival) remain sparse. Recent systematic reviews highlight this gap; for instance, a 2024 review by Khatri et al. on pediatric populations found limited RCT evidence for genotype-informed Bayesian dosing, calling for more robust trial designs [68]. Consequently, the most compelling current evidence supports the use of Bayesian methods for improving pharmacokinetic target attainment, a key surrogate endpoint, while definitive proof of impact on long-term hard clinical outcomes awaits further RCT data.

Yogesh et al. explored a hybrid artificial intelligence framework combining statistically equivalent signatures (SES), LASSO-based feature selection, and ensemble deep learning models based on long short-term memory (LSTM) and gated recurrent unit (GRU) architectures [70]. Although this work focused on chronic kidney disease classification rather than direct dose prediction, it is methodologically relevant to immunosuppressant dosing because it addresses feature redundancy and model robustness in high-dimensional clinical datasets. The authors demonstrated that multiple covariate subsets could yield statistically equivalent predictive performance, a finding that mirrors challenges in pharmacokinetic modelling where numerous correlated clinical variables are available. Their ensemble LSTM–GRU model achieved approximately a 2% absolute improvement in classification accuracy compared with traditional machine learning methods such as logistic regression, decision trees, random forests, and support vector machines, with parallel improvements in precision, recall, and F1-score. Importantly, several routinely collected variables contributed little to predictive performance, highlighting that more data do not necessarily translate into better predictions. However, the lack of external validation and the focus on classification rather than continuous exposure or dose outcomes limit the direct clinical translatability of these findings to immunosuppressant dosing.

More direct evidence for the clinical value of MIPD is provided by Greppmair et al., who performed a large-scale external evaluation of 24 published population pharmacokinetic models for piperacillin in critically ill patients [71]. Using data from 561 ICU patients and 3654 plasma concentrations, the authors quantified how Bayesian forecasting markedly improves predictive performance. A priori model predictions without therapeutic drug monitoring showed substantial variability, with median prediction errors ranging from −135.6% to +78.3% and median absolute prediction errors between 35.7% and 135.6%. After incorporating a single concentration measurement into Bayesian updating, the median absolute prediction error improved by approximately 45%, while the inclusion of two concentrations resulted in a 67.5% improvement. Precision also improved substantially, with median absolute prediction error reductions of 29% and 39% after one and two samples, respectively. The best-performing model reduced bias from −9.8% in a priori predictions to −0.9% after Bayesian updating, with a corresponding decrease in median absolute prediction error from 37% to 23.7%. These quantitative improvements illustrate how Bayesian approaches reduce inter- and intra-patient variability and enhance predictive accuracy, findings that are highly relevant for immunosuppressants such as tacrolimus, where early post-transplant exposure control is critical.

The numerical reliability of Bayesian dosing platforms was further demonstrated by Ravix et al., who conducted a comprehensive verification study of the Tucuxi Bayesian software against NONMEM using simulated datasets comprising 4000 virtual patients [72]. Across a range of pharmacokinetic scenarios, including oral and intravenous administration and both sparse and rich sampling designs, the agreement between Tucuxi and NONMEM was exceptionally high. In 99.8% of cases, relative prediction errors were below 0.1%, with a mean prediction error of 0% (range −0.09% to 0.07%) and a relative root mean squared error of 0.82%. Bioequivalence criteria for key exposure metrics, including AUC_0_–_24_, Cmin, and Cmax, were satisfied in all evaluated models, with median equivalence ratios of 100%. While this study did not assess clinical outcomes such as time to therapeutic range or rejection rates, it provides strong quantitative evidence that modern Bayesian tools can deliver numerically robust and reproducible exposure predictions suitable for clinical implementation.

Large-scale real-world evidence of clinical benefit was reported by Labriffe et al., who analyzed 3632 mycophenolic acid AUC estimations from 1872 liver transplant recipients over a 15-year period using Bayesian dose adjustment [67]. At the initial assessment, patients were frequently underexposed, with a median AUC_0_–_12_ of 27 mg·h/L (interquartile range 16–39), below the recommended therapeutic window of 30–60 mg·h/L. Following Bayesian-guided dose individualisation, median AUC_0_–_12_ increased significantly to 38 mg·h/L (interquartile range 30–49; *p* < 0.001). Most notably, the proportion of patients within the therapeutic range increased from 40% to 61%, representing a 21% absolute improvement in target attainment. Although formal variability metrics were not reported, the substantial increase in on-target exposure strongly suggests a reduction in clinically relevant inter-patient variability.

Several specialized software platforms have been developed to facilitate Bayesian dosing in clinical environments. Programmes such as BestDose, MW/Pharm++, InsightRx, and NextDose incorporate established population pharmacokinetic models and allow real-time Bayesian forecasting based on patient-specific inputs [73]. These tools typically require the clinician to enter demographic and laboratory data, along with measured drug concentrations, after which the software calculates individualized pharmacokinetic parameters and suggests the next optimal dose. Cloud-based systems like *InsightRx Nova™* extend these capabilities by integrating directly with electronic health records (EHRs), enabling automatic data retrieval and seamless clinical workflow integration [60]. Such platforms provide not only numerical dosing recommendations but also visual dashboards displaying predicted concentration–time profiles, uncertainty intervals, and the expected impact of alternative dosing scenarios.

The adaptability of Bayesian models is particularly advantageous during the unstable post-transplant phase, where fluctuations in renal function, hepatic metabolism, haematocrit, and drug interactions can rapidly alter pharmacokinetics. By continuously updating parameter estimates, Bayesian forecasting can detect emerging deviations from expected kinetics and recommend timely dose adjustments before toxicity or rejection occurs [74]. This proactive capacity aligns closely with the goals of precision medicine—anticipating rather than reacting to variability.

Moreover, Bayesian modelling provides a transparent and mechanistically interpretable framework that fosters clinician trust. Each prediction is accompanied by an estimate of uncertainty, allowing the physician to balance therapeutic decisions according to clinical judgement and patient-specific risk tolerance. Unlike black-box AI algorithms, Bayesian methods preserve a clear physiological rationale: the model’s structure directly corresponds to known pharmacokinetic processes such as absorption, distribution, metabolism, and elimination. This interpretability makes Bayesian models particularly attractive in regulatory and clinical contexts, where transparency and reproducibility are essential.

Recent developments have begun to merge Bayesian pharmacokinetics with advanced machine learning methods, creating hybrid or hierarchical models that combine the strengths of both approaches. In these systems, machine learning algorithms can enhance prior distributions by identifying latent patterns across large datasets, while Bayesian updating preserves individual-level interpretability and statistical rigour. For example, population priors for tacrolimus clearance can be dynamically recalibrated based on evolving institutional data, genotype frequencies, or patient demographics [75]. This synergy between AI and Bayesian modelling allows for both scalability and personalisation—learning from broad population trends while tailoring predictions to the unique physiological context of each patient. A key application of this synergy is the creation of population-informed or genotype-stratified priors. To address inter-population variability, Bayesian models can incorporate distinct a priori pharmacokinetic parameter distributions (e.g., for clearance and volume of distribution) that are conditional on a patient’s pharmacogenotype or inferred genetic ancestry. For instance, a model may use one set of population mean parameters for CYP3A5 *1/*1 (rapid metabolizers), another for *1/*3 (intermediate), and a third for *3/*3 (poor metabolizers). When genotype is unavailable, prior distributions can be weighted based on the known allele frequencies of the patient’s demographic group. In a hybrid Bayesian-AI framework, machine learning can further refine these priors by identifying latent covariates from real-world data that correlate with genetic subgroups, allowing for dynamic prior adaptation as a model is deployed in a new clinical setting. This approach moves beyond a one-size-fits-all population prior towards a library of priors that reflect the genetic and physiological diversity of global transplant recipients.

In parallel, the integration of Bayesian forecasting into digital health infrastructure is transforming how therapeutic drug monitoring is conducted. With cloud-based computation and point-of-care connectivity, Bayesian models can now operate in near real time. A clinician can input a patient’s latest concentration data, and within seconds, the system recalculates individualized pharmacokinetic parameters and proposes a new dosing regimen. This immediacy facilitates data-driven clinical decision-making during rounds or telemedicine consultations and supports consistent, standardized dosing across multidisciplinary transplant teams. Some platforms are even incorporating adaptive feedback loops in which each implemented dosing decision further refines the institutional model, creating a learning healthcare system that continuously improves over time.

The evidence supporting Bayesian-guided dosing in transplantation continues to expand. Clinical trials and observational studies have consistently demonstrated improved outcomes in patients managed with Bayesian prediction models, including reduced variability in trough levels, fewer supratherapeutic exposures, and shorter times to reach stable dosing. Beyond tacrolimus, similar benefits have been reported for mycophenolate mofetil, cyclosporine, and everolimus, suggesting that the Bayesian paradigm is broadly applicable across immunosuppressant classes [76,77]. As computational resources and digital infrastructures become more accessible, it is increasingly feasible for transplant centres of varying sizes to adopt these systems as part of routine care.

Nevertheless, challenges remain in widespread implementation. Successful use of Bayesian models requires validated population priors that accurately represent the target population, standardized laboratory measurements to ensure data reliability, and clinician training to interpret and act upon model outputs appropriately.

**Figure 1:**
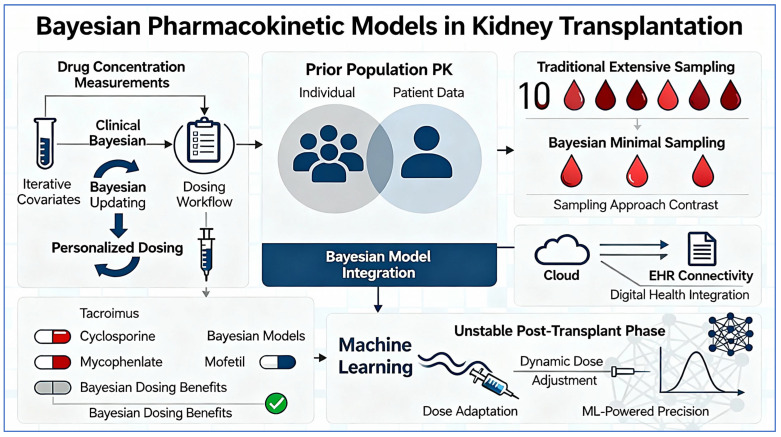
Bayesian Pharmacokinetic Models in Kidney Transplantation.

Figure 1 illustrates the Bayesian pharmacokinetic workflow in kidney transplantation, highlighting how population priors, patient-specific covariates, and therapeutic drug monitoring data are integrated to generate individualized posterior predictions. The figure demonstrates the iterative feedback loop through which each new concentration measurement refines pharmacokinetic parameter estimates and informs subsequent dose recommendations.

## 6. Digital Tools and Clinical Decision-Support Platforms

Implementation of artificial intelligence and Bayesian pharmacokinetic approaches in clinical environments has progressed through the development of specialized digital platforms and decision-support systems that enable precision dosing during patient care. Such digital tools form the practical foundation for precision pharmacotherapy in transplantation settings, serving as interfaces between computational predictions and clinical treatment decisions. By integrating pharmacokinetic modelling, electronic health record (EHR) data, and user-friendly interfaces, digital tools have transformed the once complex process of model-informed precision dosing into a routine component of clinical care. Their rapid evolution exemplifies how digital health and computational pharmacology can converge to support individualized immunosuppression management in kidney transplantation.

Modern decision-support systems for immunosuppressant dosing can be broadly classified into three functional categories: pharmacometric software platforms, cloud-based decision-support environments, and mobile or patient-facing digital applications. Based on the predictions, the system can recommend optimal dosing regimens to achieve target exposure [78]. Most of these platforms provide graphical representations of concentration–time profiles, 95% prediction intervals, and the expected consequences of alternative dosing strategies [79]. Such visual tools enhance clinician understanding and confidence, facilitating communication with patients and multidisciplinary teams.

Cloud-based decision-support systems have further advanced the integration of predictive modelling into everyday clinical workflows. These platforms, often embedded directly within hospital EHR systems, allow real-time synchronization of laboratory data, pharmacogenomic profiles, and dosing history. For example, *InsightRx Nova™* and *DoseMeRx* use population pharmacokinetic priors in combination with Bayesian updating to generate individualized dosing recommendations that can be automatically documented in the patient’s record [80,81]. These systems minimize transcription errors, reduce turnaround times, and ensure standardized decision-making across clinicians and shifts. Moreover, the scalability of cloud-based architectures enables multi-institutional data aggregation, allowing predictive models to learn continuously from a growing repository of patient outcomes. This creates a self-improving, data-driven ecosystem—a hallmark of a learning healthcare system, where each clinical interaction contributes to collective model refinement.

Digital tools are increasingly leveraging machine learning and artificial intelligence to enhance their predictive accuracy and adaptability. Machine learning algorithms embedded within decision-support platforms can analyze cumulative data streams from thousands of transplant recipients, identifying population trends, latent risk factors, and previously unrecognized sources of pharmacokinetic variability. Over time, such models evolve from static predictors into adaptive systems capable of recalibrating themselves as new evidence emerges. Importantly, these platforms can integrate pharmacogenomic and laboratory data to offer genotype-guided dosing in real time. For instance, *AID-Tac,* an AI-based dosing system developed specifically for tacrolimus, uses supervised learning algorithms to predict the optimal initial dose based on CYP3A5 genotype, demographic parameters, and early therapeutic monitoring results [82]. This integration of genomics and artificial intelligence has already demonstrated improved prediction accuracy and reduced variability in achieving target tacrolimus levels, underscoring the potential of such tools in clinical transplantation.

Mobile health (mHealth) applications have extended precision dosing beyond the clinic, empowering patients to participate actively in their therapeutic management. These applications can track medication adherence, dosing times, and side effects, transmitting real-time data to the clinical team. Some systems also incorporate reminders, symptom diaries, and integration with wearable devices that monitor physiological parameters such as heart rate, blood pressure, and renal function indicators [83]. By linking these patient-generated data streams with centralized Bayesian or AI-based dosing platforms, clinicians can monitor adherence and adjust therapy dynamically. This bidirectional communication strengthens continuity of care, improves adherence rates, and reduces the incidence of sub-therapeutic exposure due to missed or delayed doses. The development and evaluation of such patient-facing tools necessitate active patient and public involvement (PPI) to ensure they are truly patient-centred. Co-design with patients and caregivers is critical to create interfaces that are intuitive, to address practical barriers to use (e.g., health literacy, digital access), and to prioritize features that align with patient-valued outcomes, such as reducing treatment burden or empowering self-management. Furthermore, PPI in the research phase ensures that trial designs and outcome measures reflect what is meaningful to the end-user, moving beyond purely clinical metrics to include patient-reported experiences and quality of life.

A key feature of modern decision-support tools is interoperability, the ability to communicate seamlessly with EHRs, laboratory information systems, and pharmacy databases. Interoperability ensures that dosing recommendations are based on the most current and comprehensive data available, reducing manual data entry and potential for error. It also allows integration of diverse data types, pharmacogenomic test results, comorbidity indices, and time-stamped medication logs into a unified analytical framework.

Several studies have demonstrated the tangible clinical benefits of digital decision-support platforms in transplantation [84,85,86]. Implementations of Bayesian and AI-assisted dosing systems have resulted in shorter times to achieve therapeutic concentrations, fewer dose modifications, reduced interindividual variability, and decreased rates of acute rejection and nephrotoxicity [58,87]. Moreover, these systems enhance workflow efficiency by automating data analysis and documentation, freeing clinicians to focus on clinical interpretation and patient interaction. In economic terms, precision dosing platforms have been associated with reduced hospital length of stay, fewer readmissions, and lower overall treatment costs by preventing dose-related complications.

These approaches have been associated with improved attainment of therapeutic drug concentrations and reduced interindividual variability, which are considered important surrogate markers for optimizing immunosuppressive therapy. However, evidence demonstrating consistent reductions in acute rejection rates, long-term graft survival benefits, or cost savings from randomized controlled trials or meta-analyses remains limited.

Nevertheless, the successful adoption of digital dosing platforms depends on addressing several challenges. Data security and patient privacy are paramount, particularly when cloud-based systems process sensitive genomic and clinical information. Compliance with international data protection standards such as HIPAA (Health Insurance Portability and Accountability Act of 1996) [88] and GDPR (The General Data Protection Regulation) [89,90] is essential. Additionally, the interpretability of algorithmic recommendations remains a concern. Clinicians must understand not only what dose is being recommended, but also why it is being recommended. The incorporation of explainable AI (XAI) modules, which transparently display how input variables influence dosing decisions, can enhance user trust and regulatory compliance [91]. Equally important is user training; clinicians and pharmacists must become familiar with digital tools and confident in their application to avoid overreliance or misuse.

From a health systems perspective, the future of digital precision dosing in transplantation lies in integrated, intelligent ecosystems rather than standalone applications. The most promising vision involves seamless integration of pharmacogenomic testing, TDM data, AI-driven analytics, and clinical decision-support interfaces within a single digital infrastructure. In such a system, Bayesian models could continuously adapt as patient data accumulate, while AI modules identify evolving patterns of risk for rejection or toxicity. These insights would be communicated to clinicians through intuitive dashboards and mobile alerts, enabling proactive management rather than reactive correction. Over time, the integration of such systems across regional and national transplant networks could yield large-scale datasets to further refine predictive models, supporting population-level optimization of immunosuppressive therapy.

## 7. Challenges, Limitations, and Future Perspectives

Although AI, Bayesian pharmacokinetic methods, and digital decision-support systems have evolved considerably in transplantation contexts, their broad implementation in clinical practice faces multiple barriers spanning scientific, technical, regulatory, and ethical domains. Understanding and addressing these barriers is critical for realizing the full potential of precision dosing in kidney transplantation and ensuring that technological innovation translates into meaningful clinical benefit. At the same time, these challenges illuminate promising avenues for future research and system development that could redefine how immunosuppressive therapy is managed in the coming decade.

Data-related issues represent primary implementation obstacles, as both machine learning and Bayesian modelling approaches require high-quality, comprehensive, and diverse input datasets for optimal performance. Yet, in many institutions, therapeutic drug monitoring data are inconsistently collected, fragmented across different information systems, or lack standardized formats. Missing values, variable sampling times, and heterogeneity in assay methods, particularly when comparing immunoassays with mass spectrometry, can introduce biases and undermine model reliability. Furthermore, many predictive algorithms are developed from retrospective single-centre datasets that reflect specific populations or institutional practices, limiting their generalizability across geographic, ethnic, and clinical contexts. This issue is particularly pronounced in pharmacogenomic research, where allele frequencies for genes such as *CYP3A5*, *ABCB1*, and *UGT1A9* differ substantially among populations [92,93]. The absence of ethnically diverse, multicentre datasets hinders the creation of universally applicable dosing models and underscores the urgent need for collaborative, international data-sharing initiatives (Table 3).

Closely related to data limitations is the challenge of model interpretability and transparency. Many ML algorithms function as complex “black boxes,” making it difficult for clinicians to understand how predictions are derived or which variables most strongly influence the recommended dose. This lack of transparency can lead to scepticism, reluctance to adopt AI-based systems, and ethical concerns regarding accountability in medical decision-making. In contrast, Bayesian models provide interpretable probabilistic reasoning but may still require specialized training to understand uncertainty intervals and posterior distributions (Table 3).

Regulatory and ethical considerations present additional layers of complexity. The integration of AI and pharmacogenomics tools into clinical practice demands rigorous validation, standardization, and compliance with healthcare regulations. Currently, there is a lack of universally accepted frameworks for the clinical evaluation of algorithmic dosing systems [94,95]. Questions persist regarding model certification, liability in case of adverse outcomes, and data privacy, particularly when using cloud-based or cross-border digital infrastructures. The handling of genomic data raises further ethical concerns, including consent, confidentiality, and potential discrimination based on genetic risk factors. Addressing these issues will require coordinated action by regulatory bodies, healthcare institutions, and developers to establish transparent validation pipelines, audit trails, and ethical oversight mechanisms tailored to AI-driven pharmacotherapy (Table 3).

The implementation of digital decision-support tools in real-world clinical workflows also encounters logistical and organizational barriers. A significant evidence gap concerns the empirical metrics of implementation itself. While pharmacokinetic outcomes are frequently reported, data on cost-effectiveness (e.g., cost per quality-adjusted life-year, QALY), detailed workflow integration time, required training hours, and clinician adoption rates are scarce. Prospective implementation studies measuring these outcomes are essential to inform health system investment and design effective roll-out strategies.

Integration with existing electronic health record systems, laboratory information networks, and pharmacy databases requires significant infrastructural investment and interoperability standards [95]. Many hospitals, particularly in low- and middle-income countries, lack the technological capacity or financial resources to deploy advanced predictive platforms at scale [96]. Furthermore, there remains a shortage of trained personnel capable of managing, interpreting, and maintaining these systems. Education and multidisciplinary collaboration between clinicians, pharmacists, geneticists, data scientists, and informaticians are essential for bridging this gap. Incorporating pharmacogenomics and AI literacy into medical and pharmacy curricula could cultivate a new generation of healthcare professionals equipped to navigate data-driven clinical environments [97].

Empirical data on cost-effectiveness, training requirements, and real-world feasibility of AI and Bayesian dosing platforms remain limited. Most studies report pharmacokinetic outcomes rather than health economic metrics. Prospective implementation studies measuring cost per quality-adjusted life-year (QALY), workflow integration time, and clinician adoption rates are needed to inform health system investment.

Addressing this human and organizational gap requires a structured approach to workforce development and training. Defined competencies in data literacy, pharmacometrics, and AI interpretation must be integrated into the continuing professional development of transplant teams. Targeted training curricula should be developed for distinct roles: clinicians need to interpret model recommendations and uncertainty intervals in the context of individual patient factors; clinical pharmacists require deeper skills in pharmacokinetic rationale and model-informed precision dosing (MIPD) software operation; and nursing staff must be proficient in data entry protocols that ensure model input quality. This training cannot be a one-time event but should be embedded in onboarding and sustained through regular simulation, case reviews, and updates as platforms evolve.

Furthermore, successful adoption depends on systematically measuring implementation outcomes beyond clinical efficacy. Prospective studies and quality improvement initiatives should evaluate acceptability and appropriateness (clinician perceptions of the tool’s relevance and fit), feasibility (the actual extent of successful implementation), fidelity (the degree to which the tool is used as intended), cost and resource utilization (the personnel time, training overhead, and infrastructure costs for sustained use), and sustainability (the tool’s integration into routine practice over the long term). Without dedicated attention to these human factors, training pathways, and implementation metrics, even technically superior platforms risk underutilization, workflow disruption, and ultimately, failure to translate predictive promise into consistent clinical benefit.

Another major limitation is the scarcity of prospective, randomized clinical trials validating the clinical efficacy and cost-effectiveness of AI-based dosing strategies compared with traditional approaches. While numerous retrospective and proof-of-concept studies have demonstrated improved predictive accuracy, evidence of long-term impact on patient outcomes, graft survival, and healthcare costs remains limited [98,99]. Furthermore, robust external validation in diverse, multi-centre cohorts, a prerequisite for establishing generalizability, is often lacking. Studies like the large-scale external evaluation of piperacillin models by Greppmair et al. (2023) demonstrate the rigorous methodological framework needed to benchmark predictive performance across real-world clinical settings before cost-effectiveness can be assessed [71]. Regulatory acceptance and clinical guideline inclusion will depend on large-scale, prospective, multicentre trials that demonstrate tangible benefits in real-world settings. Moreover, adaptive trial designs that incorporate real-time data analysis could help accelerate the evaluation and refinement of these technologies.

From a technical standpoint, future systems must evolve toward dynamic, integrative, and interoperable ecosystems capable of synthesizing multiple layers of patient data, clinical, pharmacokinetic, pharmacogenomic, proteomic, and even digital biomarkers derived from wearable devices. The convergence of these multimodal datasets will enable more accurate and context-aware dose predictions that reflect the patient’s current physiological state. Emerging fields such as digital twin modelling, where computational replicas of individual patients simulate drug behaviour and therapeutic response, offer an exciting frontier for transplant pharmacology. By continuously updating these virtual models with real-world data, clinicians could test different dosing scenarios before applying them in practice, thereby minimizing the risk of toxicity or rejection.

Artificial intelligence will also play a pivotal role in enabling adaptive and autonomous therapeutic systems. Reinforcement learning algorithms, for instance, could continuously refine dosing strategies based on real-time feedback from laboratory results and clinical outcomes. Over time, such systems may evolve into semi-autonomous therapeutic control loops, operating under clinician supervision but capable of making rapid, data-driven adjustments to dosing regimens. The integration of these technologies into smart infusion pumps, implantable biosensors, or mobile applications could usher in an era of closed-loop pharmacotherapy, where drug delivery adapts continuously to changing physiological and environmental conditions (Table 4).

Moving from principles to practice requires delineating concrete regulatory pathways and consent frameworks. For regulatory approval, AI-based dosing platforms typically fall under the classification of Software as a Medical Device (SaMD) or Clinical Decision Support Systems (CDSS). In the United States, the U.S. Food and Drug Administration’s (FDA) Digital Health Center of Excellence provides guidance, with many tools seeking approval via the 510(k) or De Novo pathways, contingent on demonstrating substantial equivalence or safety and effectiveness for novel devices. The European Union’s Medical Device Regulation (MDR) and the newly enacted AI Act, which classifies high-risk medical AI systems under strict transparency and robustness requirements, create a parallel yet distinct framework. A key challenge is the pre-market validation of adaptive AI systems that continue to learn post-deployment, necessitating novel regulatory approaches for real-world performance monitoring and change control protocols.

Regarding genomic data governance, the integration of pharmacogenomics into AI models demands consent models that go beyond traditional clinical data agreements. Dynamic or tiered consent frameworks are particularly relevant, allowing patients to specify and update their preferences regarding future research use, data sharing across institutions, and participation in algorithm training. Consent processes must clearly differentiate between using genetic data for immediate clinical care (e.g., CYP3A5-guided initial dosing) and for secondary research purposes such as refining population pharmacokinetic priors or training machine learning models. Governance structures, such as dedicated Data Access Committees (DACs) and Algorithmic Review Boards, are essential to steward these sensitive datasets, ensure compliance with the General Data Protection Regulation (GDPR) principles of purpose limitation and data minimization, and manage ethical dilemmas like the handling of incidental findings. Technical solutions like federated learning, where model training occurs across decentralized data sources without centralizing raw genomic information, offer a promising path to advancing predictive models while mitigating privacy risks and logistical barriers to data sharing.

Looking ahead, the future of precision dosing in transplantation lies in collaborative intelligence, the synergistic partnership between human expertise and computational power. AI and Bayesian models are not designed to replace clinical judgement but to enhance it, providing clinicians with objective, data-rich insights that support decision-making. Achieving this vision will require a cultural shift in medicine, emphasising trust, transparency, and continuous learning. Institutions must foster environments where algorithmic recommendations are critically evaluated, refined through experience, and integrated into shared clinical reasoning.

The establishment of global pharmacometric networks and open-access data repositories represents another essential step forward. Collaborative platforms that pool anonymized TDM and pharmacogenomic data from transplant centres worldwide could dramatically expand the diversity and robustness of model training datasets. Such initiatives would not only improve predictive accuracy but also facilitate equitable access to precision dosing tools in resource-limited settings. International partnerships between academic institutions, healthcare providers, regulatory agencies, and industry could accelerate the development of standardised, validated, and ethically governed digital infrastructures for model-informed precision dosing.

Comparatively, machine learning models excel in capturing complex nonlinear relationships and high-dimensional interactions, particularly when large datasets are available, but may suffer from limited interpretability and external generalizability (Table 5). Bayesian pharmacokinetic models offer mechanistic transparency, robust performance with sparse data, and easier clinical integration, though they rely on well-validated population priors. Compared with traditional trough-based dosing, both ML- and Bayesian-guided approaches have a stronger evidence base, consistently demonstrating improved exposure control, reduced variability, and faster dose stabilisation in observational and simulation studies. In contrast, claims regarding the superiority of hybrid Bayesian-AI models remain largely speculative, as noted in Table 5. While the conceptual synergy between mechanistic and data-driven approaches is compelling, there is a current absence of reproducible clinical implementations, open-source code, standardised benchmarks, and prospective validation to substantiate performance advantages over established Bayesian or standalone ML methods. Therefore, hybrid approaches represent an important investigational path forward, but their translation into clinical practice requires a shift from proof-of-concept studies to rigorous, head-to-head comparative evaluations with transparent reporting of code and model performance metrics.

## 8. Translational Research and Actionable Clinical Guidance

To translate this potential into widespread clinical benefit, specific and actionable steps are necessary. For clinicians and transplant centres, the immediate priority should be to adopt and develop familiarity with Bayesian model-informed precision dosing (MIPD) platforms, which represent the most mature and evidence-supported tools for advancing beyond trough-guided monitoring. Implementing these systems requires dedicated training for multidisciplinary teams to ensure clinicians, pharmacists, and nursing staff can effectively interpret model outputs and their associated uncertainty. Currently, AI-driven dosing tools are best utilized within structured research or quality improvement initiatives to build local evidence and clinical experience.

Second, implementation science studies are urgently needed to move beyond efficacy to practical effectiveness. These studies should quantify the resources required for sustained use, evaluate training pathways for multidisciplinary teams, identify workflow integration barriers, and measure fidelity of use in real-world settings across diverse healthcare environments, including resource-limited contexts.

For researchers, efforts must be directed toward conducting prospective, pragmatic, multicenter trials that compare advanced dosing strategies, such as Bayesian, machine learning, and hybrid models, against standard care. These trials should prioritize hard clinical endpoints, including biopsy-proven acute rejection and graft survival, as well as implementation outcomes like feasibility, cost, and workflow integration. The highest research priority is to conduct head-to-head comparisons of established Bayesian methods against newer AI approaches. Furthermore, all model development studies must adhere to FAIR principles (Findable, Accessible, Interoperable, Reusable), with public sharing of code and validation against external datasets to ensure reproducibility and broad generalizability.

For regulators and guideline developers, there is a critical need to establish harmonized frameworks for the evaluation and certification of adaptive dosing algorithms. Clinical guidelines should be updated to formally recognize MIPD based on Bayesian forecasting as a recommended strategy for managing drugs with narrow therapeutic indices, such as tacrolimus [16]. Concurrently, clear regulatory pathways must be created to facilitate the prospective validation and safe introduction of AI-based tools into clinical practice.

A focused and prioritized research agenda is essential. The primary question is whether Bayesian or AI-guided dosing protocols improve graft survival and patient outcomes compared to standard therapeutic drug monitoring in randomized controlled trials. Second, direct comparative effectiveness research must determine if hybrid or pure ML models offer superior clinical or operational performance compared to well-implemented Bayesian MIPD. Third, implementation science studies are needed to identify the optimal strategies for training, workflow integration, and sustaining these technologies across diverse healthcare settings, including resource-limited environments. Finally, research must address equity and generalizability by determining how models and platforms can be designed and validated to ensure equitable performance across diverse genetic ancestries and clinical phenotypes.

By combining rigorous clinical validation with thoughtful implementation and a prioritized research agenda, the field can ensure that the promise of predictive precision dosing leads to tangible improvements in the longevity of kidney transplants and the safety of patients.

## 9. Conclusions

AI and predictive modelling approaches offer promising avenues for transforming immunosuppressant dosing precision in kidney transplantation settings. Machine learning can uncover complex, non-linear dose–response relationships, while Bayesian pharmacokinetic models provide an interpretable and adaptive framework for individualisation. Achieving this potential requires transitioning from preliminary demonstration studies to systematically implemented research and clinical application programmes. In the short term (1–3 years), the highest priority is to conduct multi-centre randomized controlled trials that definitively establish the efficacy of model-informed precision dosing (MIPD) using robust clinical endpoints like graft survival and rejection. Concurrently, standardized reporting guidelines for such studies must be established. In the medium term (3–5 years), research should pivot to implementation science, identifying optimal training, workflows, and cost-effective deployment models, and to head-to-head comparisons evaluating whether advanced AI offers a measurable advantage over validated Bayesian methods. Underpinning this entire effort must be a foundational commitment to equity. This requires validating tools in diverse populations, developing resource-appropriate versions for low-resource settings, and fostering collaborative, open-science ecosystems. By pursuing this prioritized path, first proving efficacy and then optimizing implementation within an equitable framework, computational advances can finally translate into safer, more effective, and more accessible care for all transplant recipients.

## Figures and Tables

**Table 1:** Sources of Variability in Immunosuppressive Therapy and the Rationale for Precision Dosing.

Category of Variability	Key Contributing Factors	Impact on Drug Pharmacokinetics (PK)/ Pharmacodynamics (PD)	Clinical Consequence
Pharmacogenomic	CYP3A5 Polymorphisms: *1 carriers (rapid metabolizers) vs. *3/*3 (poor metabolizers).	Significant inter-patient differences in drug metabolism and clearance. Highly variable systemic exposure from identical doses.	High risk of subtherapeutic exposure (rejection) or supratherapeutic exposure (toxicity) with standard dosing.
	ABCB1 (P-glycoprotein) Variants: Alters drug transport (absorption, distribution).		
	UGT1A9 and SLCO1B1 Polymorphisms: Affects mycophenolate metabolism.		
	CYP3A4 Variants: Influences mTOR inhibitor clearance.		
Physiological and Clinical	Demographics: Age (pediatric/elderly), body composition.	Dynamic, time-dependent changes in drug distribution, protein binding, and clearance, especially post-transplant.	Unstable drug levels; constant dose adjustments are required to maintain the therapeutic window.
	Organ Function: Fluctuating renal/hepatic function, haematocrit, albumin.		
	Drug–Drug Interactions: CYP3A inhibitors/inducers.		
	Clinical State: Inflammation, infection.		
Behavioural and Environmental	Medication Non-Adherence: Due to complex regimens.	Unpredictable and variable drug absorption and bioavailability. Altered enzyme activity.	Erratic drug exposure; complicates dose optimization and is a leading cause of late graft loss.
	Diet and Timing: Food intake (e.g., high-fat meals), circadian rhythm.		
	Lifestyle: Herbal supplements, smoking.		
Analytical and Methodological (TDM Limitations)	Trough (C_0_) Monitoring: A single, static data point.	Incomplete picture of total drug exposure over time. Potential for inaccurate PK parameter estimation.	Dose adjustments based on incomplete or potentially misleading information, increasing the risk of incorrect dosing.
	AUC Monitoring: Logistically challenging (multiple samples).		
	Assay Differences: Immunoassay vs. LC-MS/MS discrepancies.		

**Table 2:** Conceptual comparison of modelling approaches used in precision dosing of immunosuppressants.

Aspect	Population Pharmacokinetics (PopPK)	Bayesian Modelling	Machine Learning (ML)	Explainable Artificial Intelligence (XAI)
Primary purpose	Describe drug PK behaviour at the population level	Individualize predictions using prior knowledge and patient data	Predict outcomes or dose requirements from data	Improve transparency and interpretability of ML models
Modelling paradigm	Mechanistic or semi-mechanistic statistical modelling	Probabilistic inference framework applied to PopPK	Data-driven, algorithmic modelling	Interpretability layer, not a standalone model
Use of prior knowledge	Yes (fixed structural and variability parameters)	Yes (explicit priors updated with individual data)	No explicit priors required	Not applicable
Individualization	Limited (population averages with covariates)	High (posterior individual estimates)	High (learned from individual-level data patterns)	Not applicable
Data requirements	Moderate (PK samples, covariates)	Moderate (PopPK model + TDM data)	High (large, diverse datasets)	Depends on underlying ML model
Interpretability	High (physiological meaning of parameters)	High (clinically transparent updating process)	Often low (“black-box” models)	High (methods to explain ML predictions)
Handling nonlinearity and complex interactions	Limited	Moderate	High	Not applicable
Typical clinical use	Model development, simulation, dose guidelines	Therapeutic drug monitoring and adaptive dosing	Dose prediction, risk stratification, outcome forecasting	Supporting trust and adoption of ML systems
Examples in transplantation	Tacrolimus PopPK models	Bayesian forecasting in InsightRx Nova™	ML-based tacrolimus dose prediction models	SHAP, feature importance for ML dosing tools
Regulatory acceptance	Well established	Well established	Emerging	Emerging

**Table 3:** Summary of Machine Learning Approaches for Immunosuppressant Dose Prediction.

ML Approach/ Category	Key Examples	Principle and Strengths	Application in Immunosuppressant Dosing	Representative Performance Outcomes	Limitations
Ensemble Learning	Random Forests	Combines multiple decision trees to capture nonlinear relationships and complex feature interactions; robust to noise and missing data	Widely applied to tacrolimus trough concentration and dose prediction using demographic, laboratory, and pharmacogenomic variables (e.g., CYP3A5 genotype)	RMSE typically 1.8–2.5 ng/mL for tacrolimus trough prediction; ~15–30% reduction in prediction error compared with linear regression when genotype data included (where reported)	Quantitative comparison across studies is limited by heterogeneous target variables (dose vs. concentration), differing sampling times, and centre-specific datasets
Gradient Boosting	XGBoost, LightGBM	Sequentially builds weak learners to minimize prediction error; performs well with imbalanced datasets and outliers	Prediction of tacrolimus dose requirements and trough levels, particularly in early post-transplant period	Frequently reported to outperform random forests and linear models in internal validation; RMSE values comparable or slightly lower than RF models in single-centre studies	Performance metrics often reported only in internal validation; lack of standardized external validation prevents robust cross-study comparison
Deep Learning	Artificial Neural Networks (ANNs)	Learns hierarchical representations of complex nonlinear relationships without explicit feature engineering	Used for predicting tacrolimus concentrations from high-dimensional clinical and laboratory data	Improved predictive accuracy over regression models in several studies; quantitative metrics vary widely depending on network architecture and input features	Limited interpretability; substantial heterogeneity in model design and reporting makes quantitative comparison across studies infeasible
Recurrent Neural Networks	RNNs	Models sequential dependencies in time-series data	Applied to longitudinal TDM data to predict future tacrolimus concentrations	Demonstrated improved temporal prediction compared with static models in pilot studies	Mostly evaluated in small datasets; lack of standardized outcome metrics and prospective validation
Long Short-Term Memory Networks	LSTMs	Specialized RNNs capable of learning long-term temporal dependencies	Particularly useful in early post-transplant phase with rapidly changing pharmacokinetics	Shown to better capture time-dependent changes in drug exposure than non-temporal models	Quantitative performance varies widely; direct comparison limited by different prediction horizons and sampling schemes
Reinforcement Learning (RL)	N/A (custom implementations)	Learns optimal dosing policies through interaction with simulated or real patient data	Adaptive dose titration based on sequential feedback from TDM results	Pilot studies report faster achievement of therapeutic range and reduced variability	Early-stage research; small cohorts, simulated environments, and lack of standardized metrics preclude quantitative comparison
Hybrid Models	Population PK + ML	Combines mechanistic PK structure with ML modelling of residual variability	Tacrolimus and cyclosporine dose prediction using sparse sampling	Reported improvements in predictive accuracy and generalizability over standalone PK or ML models	Performance gains vary by implementation; limited number of studies and inconsistent reporting of quantitative metrics
Bayesian + ML	Bayesian PK with ML-enhanced priors	ML informs or adapts Bayesian priors while preserving interpretability	Individualized dosing with dynamic updating as new data accrue	Improved stability of predictions and reduced uncertainty reported qualitatively	Quantitative comparison difficult due to diverse Bayesian structures and ML components
Explainable AI (XAI)	SHAP, LIME	Provides interpretability by quantifying feature contributions to predictions	Enhances clinician trust by explaining ML-based dosing recommendations	Does not directly improve predictive performance; supports clinical adoption	Not a predictive model itself; quantitative performance metrics not applicable

**Table 4:** Challenges, Limitations, and Future Perspectives in the Field of Kidney Transplantation.

Challenge Category	Description	Clinical Impact	Technical/Operational Issues	Future Directions
Data Quality and Availability	Incomplete, fragmented, and heterogeneous therapeutic drug monitoring data. Lack of standardised formats and sampling variability. Retrospective, single-centre datasets limit generalizability.	Limits model accuracy and predictive reliability; may produce biased dosing recommendations.	Data fragmentation across systems; assay variability; missing or inconsistent data.	Development of multinational, ethnically diverse real-world databases; standardized TDM protocols; data sharing networks.
Model Interpretability	Machine learning often forms “black boxes” lacking transparency; Bayesian models provide probabilistic but complex outputs.	Reduced clinician trust and adoption; ethical concerns over accountability.	Need for specialized training to interpret uncertainty estimates in Bayesian outputs.	Training programmes; development of intuitive interfaces; explainable AI approaches to enhance transparency.
Regulatory and Ethical Issues	Lack of harmonized regulatory frameworks for AI/PK models; concerns about liability, certification, privacy, and genomic data handling.	Barriers to clinical approval and integration; potential legal and ethical risks.	Diverse regulatory environments; complex liability and data governance issues; genomic data sensitivity.	Establishment of international regulatory standards; robust audit trails and ethical oversight mechanisms.
Clinical Integration	Difficulty embedding tools into existing workflows; infrastructure gaps and costs; shortage of trained personnel.	Delayed adoption; fragmented clinical workflows; suboptimal use of models.	Interoperability challenges across EHR, labs, and pharmacies; resource constraints especially in low-income settings.	Investment in interoperable digital platforms; multidisciplinary training including AI and pharmacogenomics literacy.
Evidence Base and Trials	Limited prospective randomized trials validating clinical efficacy and cost-effectiveness of AI-based dosing methods.	Slower regulatory acceptance and guideline incorporation; lack of clinician confidence.	Need for large multicentre adaptive clinical trials integrating real-time data analysis.	Conducting prospective, multicentre, adaptive clinical trials; real-world evidence generation.
Technological Advancement	Need for integrative models synthesizing multimodal data, including digital biomarkers and physiologic states.	Potential for more precise, real-time dose adjustment aligned with patient status.	Technical complexity in data integration; need for high computational power and data standards.	Development of “digital twin” patient models; adoption of adaptive, autonomous AI dosing systems.
Collaborative Intelligence	Synergizing human clinical expertise with computational model outputs for enhanced decision-making.	Improved clinical decisions without replacing physician judgement.	Cultural and educational barriers to technology adoption; need for trust-building and iterative model refinement.	Promote clinician-AI collaboration, foster trust and transparency, and enhance continuous learning in healthcare settings.
Global Data Sharing	Need for open-access pharmacometric and pharmacogenomic data repositories to enhance model training and applicability.	Broader applicability and equity in precision dosing globally.	Data privacy concerns; cross-border legal and ethical complexities.	Develop global data-sharing frameworks, promote international collaboration and standardization.

**Table 5:** Synthesis of Evidence and Knowledge Gaps in Predictive Precision Dosing for Kidney Transplantation.

Domain	Areas of Consensus (Supported by Current Evidence)	Key Evidence Gaps	Unresolved Controversies and Research Priorities
Bayesian PopPK Modelling	Bayesian PopPK-guided dosing improves target exposure attainment compared with trough-based monitoring in observational cohorts; enables reliable AUC estimation from sparse sampling; provides mechanistic interpretability and uncertainty quantification.	Limited number of prospective randomized controlled trials; heterogeneity in outcome definitions (AUC vs. C_0_); lack of pooled effect estimates and standardized reporting.	Whether Bayesian-guided dosing translates into improved graft survival, rejection rates, or cost-effectiveness; optimal endpoints for regulatory approval.
Machine Learning-Based Models	ML models can reduce prediction error for tacrolimus concentrations in retrospective datasets; nonlinear and high-dimensional relationships are better captured than with linear regression.	Small sample sizes; inconsistent validation strategies; limited external and prospective validation; poor reproducibility due to incomplete reporting of model specifications.	Whether ML models provide clinically meaningful benefit beyond Bayesian PopPK models when evaluated head-to-head in pragmatic trials.
Hybrid Bayesian–AI Approaches	Conceptual synergy between mechanistic PK models and data-driven learning is well established; hybrid models may improve prior specification and stability.	Lack of reproducible clinical implementations; absence of open code, standardized benchmarks, and external validation datasets.	Whether hybrid models meaningfully improve clinical outcomes or primarily increase technical complexity without added benefit.
Pharmacogenomics Integration	Strong biological rationale for genotype-informed dosing (e.g., CYP3A5 for tacrolimus); incorporation into Bayesian priors is methodologically feasible.	Limited population-specific allele frequency synthesis; unclear how to dynamically adjust priors across ethnically diverse cohorts.	Whether genotype-guided priors should be static, adaptive, or population-stratified in global transplant settings.
Digital Decision-Support Platforms	Integration with EHRs enables real-time dosing recommendations and standardized workflows; platforms operationalize Bayesian dosing at the bedside.	Limited transparency of proprietary algorithms; unclear regulatory status; scarce independent clinical outcome evaluations.	Balancing automation with clinician oversight; defining liability and certification pathways for AI-driven dosing systems.
Explainable AI (XAI)	XAI tools enhance interpretability and clinician trust in ML-based predictions; useful for identifying influential covariates.	Limited evaluation of clinical utility in transplant-specific decisions; predominantly local, not global, interpretability.	Whether explainability improves decision quality or adoption, and how to standardize interpretability reporting.
Clinical Outcomes	Improved exposure metrics (AUC attainment, reduced variability) are consistently reported.	Sparse evidence linking predictive dosing to hard outcomes (rejection, graft survival, mortality).	Determining which intermediate outcomes are sufficient surrogates for long-term clinical benefit.
Ethics, Regulation, and Data Governance	Ethical concerns around transparency, accountability, and genomic data privacy are widely recognized.	Lack of concrete consent models and harmonized regulatory frameworks for AI-based dosing tools.	Defining scalable, cross-border governance models for genomic and algorithmic decision-support systems.
Implementation and Equity	Workforce training and interoperability are critical for adoption; low-resource settings face additional barriers.	Few studies report implementation metrics, costs, or training requirements.	Developing simplified, resource-appropriate dosing tools without exacerbating health inequities.

## Data Availability

No new data were created or analyzed in this study.
